# T Cell Expression of Granulocyte–Macrophage Colony-Stimulating Factor in Juvenile Arthritis Is Contingent Upon Th17 Plasticity

**DOI:** 10.1002/art.38647

**Published:** 2014-06-27

**Authors:** Christopher Piper, Anne M Pesenacker, David Bending, Balathas Thirugnanabalan, Hemlata Varsani, Lucy R Wedderburn, Kiran Nistala

**Affiliations:** University College LondonLondon, UK

## Abstract

**Objective:**

Granulocyte–macrophage colony stimulating factor (GM-CSF) is a potent inflammatory mediator that is responsible for recruitment and activation of innate immune cells. Recent data from murine studies have identified Th17 cells as a key source of GM-CSF and suggest that T cell–derived GM-CSF is instrumental in the induction of autoimmune disease. The present study was undertaken to analyze the expression of T cell–derived GM-CSF in the joints of patients with juvenile idiopathic arthritis (JIA) and to investigate the differentiation of Th17 cells and how this relates to GM-CSF+ T helper cells.

**Methods:**

Synovial fluid (SF) and peripheral blood (PB) samples from 24 patients with JIA were analyzed, by flow cytometry and reverse transcription–polymerase chain reaction, for expression of GM-CSF and the Th17 marker CD161. A cytokine capture assay was used to purify Th17 cells and test the plasticity of cytokine production in response to interleukin-12 (IL-12) and IL-23.

**Results:**

The frequency of GM-CSF–producing T helper cells was significantly enriched in SF mononuclear cells compared to PB mononuclear cells from the patients with JIA (24.1% of CD4+ T cells versus 2.9%) and closely correlated with the erythrocyte sedimentation rate (r^2^ = 0.91, *P* < 0.001). Synovial GM-CSF+ T cells were predominantly CD161+ and coexpressed interferon-γ (IFNγ), but not IL-17. Culture of Th17 cells in the presence of IL-12 led to rapid up-regulation of GM-CSF and IFNγ, recapitulating the phenotype of GM-CSF–expressing cells within the joint.

**Conclusion:**

Our results identify a novel outcome of Th17 plasticity in humans that may account for the enrichment of GM-CSF–expressing T cells in the joints of patients with JIA. The association of GM-CSF expression with systemic inflammation highlights the potential role of Th17-related cytokines in the pathology of JIA.

Juvenile idiopathic arthritis (JIA) is the most common form of autoimmune rheumatic disease in childhood, with a prevalence rate of 1/1,000 children under the age of 16 years (1). The successful introduction of therapies targeting tumor necrosis factor α (TNFα) has led to significant improvement in JIA outcomes. However, in one-third of patients the disease remains resistant or only partially responsive to anti-TNFα therapy, suggesting ongoing uncontrolled immunopathology that is independent of TNFα (2). The identification of a novel CD4+ T cell subset expressing interleukin-17 (IL-17) in a mouse model of arthritis led many to suggest that these cells (Th17 cells) have a role in human disease (3). We and others have demonstrated major enrichment of Th17 cells in the inflamed joints of children with JIA, with a correlation between the frequency of these cells and the severity of disease (4,5). It was therefore unexpected when data from studies of IL-17–deficient mice suggested that IL-17 was redundant for induction of autoimmunity in a mouse model of multiple sclerosis, and that granulocyte–macrophage colony-stimulating factor (GM-CSF) was instead necessary and sufficient for disease (6,7).

GM-CSF is similarly important in mouse models of arthritis and is found in high concentrations in the synovial fluid (SF) of patients with rheumatoid arthritis and JIA (8–10) It has widespread effects, promoting granulopoiesis and activating neutrophils, monocytes, and macrophages that contribute to joint inflammation and damage (9,10). Although GM-CSF is widely expressed in both stromal and hematopoietic compartments, recent murine studies suggest that GM-CSF from the hematopoietic compartment, particularly CD4+ T cells, is essential for disease (6,7,11). In mice, GM-CSF–secreting T cells are closely linked with the Th17 lineage downstream of retinoic acid receptor–related orphan nuclear receptor γt (RORγt) (murine homolog of RORC2), although data on transcriptional control of GM-CSF are conflicting (6,7). Activated human IL-17+ T cell clones produce GM-CSF (12), but the regulation of GM-CSF production in terms of response to the IL-12/IL-23 axis remains unknown, as does the exact relationship between GM-CSF– and IL-17–secreting cells. To date, evidence for the putative role of T cell–derived GM-CSF in autoimmune disease comes largely from murine studies. In the present study we examined this issue in human autoimmune arthritis.

## MATERIALS AND METHODS

### Patients and controls

Samples studied were from 24 children who met the International League Against Rheumatism criteria for JIA (13) (21 with oligoarticular disease, 3 with polyarticular disease) and 13 adult healthy controls. Seventeen of the JIA patients were female and 7 were male; the median age was 10.8 years. The study was approved by the local ethical review committee, and full informed consent was obtained from patients/parents and control subjects.

### Cell sorting and flow cytometry

Peripheral blood mononuclear cells (PBMCs) and SF mononuclear cells (SFMCs) were isolated by density centrifugation. For analysis of T cell cytokine production, cells were cultured for 4 hours in the presence of 50 ng/ml phorbol myristate acetate (PMA), 500 ng/ml ionomycin, and 5 μg/ml brefeldin A (Sigma-Aldrich) before intracellular cytokine staining as previously described (4). Antibodies against the following human proteins were used: PC7-conjugated CD4 (Beckman Coulter), phycoerythrin-conjugated CD161 (eBioscience), fluorescein isothiocyanate– or v450-conjugated interferon-γ (IFNγ) (both from BD Biosciences), Alexa Fluor 488–conjugated IL-17A (eBioscience), and Alexa Fluor 647–conjugated GM-CSF (eBioscience). A Live/Dead discriminant dye was used according to the instructions of the manufacturer (Life Technologies). To capture cytokine-expressing cells, PBMCs or SFMCs were enriched for CD4+ T cells using magnetic beads (StemCell Technologies) and stimulated for 2 hours with PMA (10 ng/ml) and ionomycin (1 μg/ml). IL-17–secreting CD4+ T cells were detected according to the instructions of the manufacturer (Miltenyi Biotec) and sorted with a flow cytometer (FACSAria; BD PharMingen). The purity of sorted cells was assessed by detection of intracellular cytokines after overnight incubation in brefeldin A. Flow cytometric data were collected on an LSRII (BD PharMingen); a minimum of 1 × 10^5^ events were collected. Data were analyzed using FlowJo (Tree Star).

### Polymerase chain reaction

RNA was extracted using TRIzol, according to the instructions of the manufacturer (Life Technologies). Generation of complementary DNA, reverse transcription—polymerase chain reaction, and analysis by the C_t_ method were performed as previously described (14). Primers used were as follows: actin forward 5′-AGA-TGA-CCC-AGA-TCA-TGT-TTG-AG-3′, reverse 5′-AGG-TCC-AGA-CGC-AGG-ATG-3′; RORC2 forward 5′-GAC-CAC-CCC-CTG-CTG-AGA-A-3′, reverse 5′-GAC-ATG-CGG-CCG-AAC-TTG-A-3′; T-bet forward 5′-CCC-CAA-GGA-ATT-GAC-AGT-TG-3′, reverse 5′-GGG-AAA-CTA-AAG-CTC-ACA-AAC-3′; and GM-CSF QuantiTect primers (Qiagen). Levels of messenger RNA (mRNA) were normalized to those of β-actin mRNA.

### Cell culture

Sorted cells were cultured in RPMI with 10% fetal calf serum (Life Technologies) in the presence of IL-2 alone or in combination with IL-12 (from R&D Systems) or IL-23 (eBioscience) (all at 10 ng/ml). On day 4, cytokine expression was detected by flow cytometry after restimulation with PMA and ionomycin in the presence of brefeldin A.

### Statistical analysis

Data were analyzed using GraphPad Prism 6. The significance of differences between group means was assessed by one-way or two-way analysis of variance, with Bonferroni correction for multiple comparisons. Correlations were assessed using Pearson's correlation coefficient. *P* values less than 0.05 were considered significant.

## RESULTS

**Figure 1:**
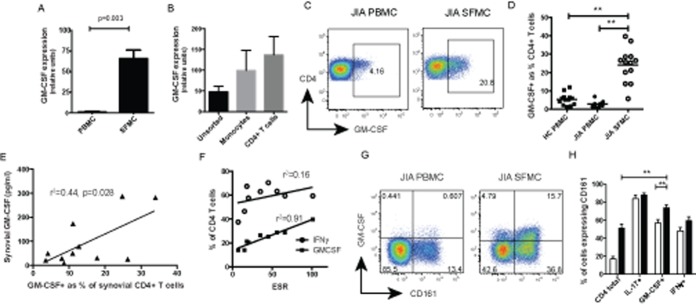
Enrichment of granulocyte–macrophage colony-stimulating factor (GM-CSF) expression within the arthritic joint. A and B, Expression of GM-CSF mRNA (normalized to mRNA for β-actin) in peripheral blood mononuclear cells (PBMCs) and synovial fluid mononuclear cells (SFMCs) from patients with juvenile idiopathic arthritis (JIA) (n = 3) (A) and in whole SFMCs, synovial CD14+ monocytes, and synovial CD4+ T cells from patients with JIA (n = 7) (B). Values are the mean ± SEM. C, Representative dot plots of GM-CSF expression, detected by flow cytometry, in JIA PBMCs and SFMCs following stimulation with phorbol myristate acetate and ionomycin in the presence of brefeldin A. All plots were gated on CD4+ T cells. D, GM-CSF expression in CD4+ T cells (detected as described in C) in healthy control (HC) PBMCs (n = 13) and in JIA PBMCs (n = 8) and SFMCs (n = 13). Symbols represent individual samples; horizontal lines show the mean. ∗∗ = *P* < 0.01 by one-way analysis of variance (ANOVA) with Bonferroni correction for multiple comparisons. E, Pearson's correlation between GM-CSF protein levels (measured by enzyme-linked immunosorbent assay) in synovial supernatants and the frequency of GM-CSF+ CD4+ T cells in paired SFMC samples. F, Pearson's correlation between the frequency of synovial interferon-γ–positive (IFNγ+) or GM-CSF+ CD4+ T cells and the serum erythrocyte sedimentation rate (ESR) (measured at the time of therapeutic arthrocentesis). G, Representative dot plots of GM-CSF and CD161 expression, detected by flow cytometry, in CD4+ T cells from JIA PBMCs and SFMCs. H, Proportion of the total CD4+ T cell population and the interleukin-17–positive (IL-17+), GM-CSF+, and IFNγ+ subsets expressing CD161 (detected as described in G) in JIA PBMCs (open bars) (n = 7) and SFMCs (solid bars) (n = 7). Values are the mean ± SEM. ∗∗ = *P* < 0.01 by two-way ANOVA with Bonferroni correction for multiple comparisons. Color figure can be viewed in the online issue, which is available at http://onlinelibrary.wiley.com/doi/10.1002/art.38647/abstract.

Historically, synovial GM-CSF production has been linked with synovial fibroblasts and innate immune cells, chiefly monocytes and tissue macrophages (9). In experiments focusing on the mononuclear cell compartment, we analyzed unsorted SFMCs directly ex vivo and detected significantly higher expression of GM-CSF mRNA in SFMCs from patients with JIA than in PBMCs from the patients (Figure 1A). To compare the contribution of T cells and monocytes, we analyzed GM-CSF mRNA expression in sorted synovial monocytes and CD4+ T cells and found that the levels were comparable (Figure 1B), reinforcing the importance of T cell–derived GM-CSF within the joint. The frequency of GM-CSF–secreting T helper cells was quantified by flow cytometry and shown to be significantly enriched within the joint (mean frequency 24.1% of CD4+ T cells) compared to PBMCs from JIA patients or healthy controls (2.9% and 5.4%, respectively) (Figures 1C and D). The frequency of GM-CSF CD4+ T cells correlated directly with levels of GM-CSF protein in SF and also with levels of inflammation as determined by the erythrocyte sedimentation rate (ESR) (r^2^ = 0.91, *P* < 0.001), whereas the frequency of IFNγ+ T helper cells in SF did not correlate with the ESR (r^2^ = 0.16*, P* = 0.2) (Figures 1E and F).

In mice, GM-CSF expression is associated with the Th17 differentiation pathway (6). This led us to investigate whether GM-CSF secretion would be limited to T cells expressing CD161, a cell surface C-type lectin-like receptor that is closely associated with human Th17 cells and RORC2 (15). We found that T cells expressing GM-CSF were enriched for CD161 compared to the total CD4+ T cell population but had lower levels of CD161 expression than IL-17+ T cells (Figures 1G and H). Consistent with our previous data (14), the proportion of T cells expressing CD161 was increased in the joint compared to levels in PBMCs, and this was also the case for GM-CSF+ T cells (Figure 1H).

**Figure 2:**
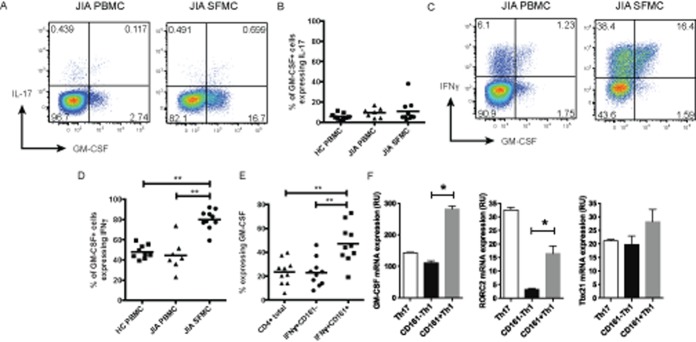
GM-CSF+ T cells share features with ex-Th17 cells. A and C, Representative dot plots of GM-CSF and IL-17 expression (A) and GM-CSF and IFNγ expression (C), detected by flow cytometry, in CD4+ T cells from JIA PBMCs and SFMCs. B and D, IL-17 (B) and IFNγ (D) coexpression (detected as described in A and C) in GM-CSF+ CD4+ T cells from healthy control PBMCs (n = 7), JIA PBMCs (n = 10), and JIA SFMCs (n = 10). E, Proportion of SFMC T cell subsets expressing GM-CSF (n = 10). In B, D, and E, symbols represent individual samples; horizontal lines show the mean. F, Expression of mRNA for GM-CSF, retinoic acid receptor–related orphan nuclear receptor C2 (RORC2), and T-bet (Tbx21) (normalized to mRNA for β-actin), as assessed by quantitative polymerase chain reaction. Th17 and Th1 subsets were identified by cytokine capture assay and purified by flow cytometry. Values are the mean ± SEM (n *=* 3). ∗ = *P* < 0.05; ∗∗ = *P* < 0.01, by one-way ANOVA with Bonferroni correction for multiple comparisons. RU = relative units (see Figure 1 for other definitions). Color figure can be viewed in the online issue, which is available at http://onlinelibrary.wiley.com/doi/10.1002/art.38647/abstract.

Given that GM-CSF+ T cells were enriched within the CD161+ cell compartment, we predicted that they may coexpress IL-17. Surprisingly, only 10% of GM-CSF+ cells were found to be IL-17+ (Figures 2A and B). In contrast, GM-CSF expression showed much greater concordance with IFNγ expression, and this association was most prominent in synovial T cells; a mean of 80.1% of GM-CSF+ CD4+ T cells coexpressed IFNγ (Figures 2C and D). Based on these findings, we wondered whether GM-CSF could be up-regulated within the “ex-Th17” cell population. We have previously shown that Th17 cells within the joint undergo plasticity toward the Th1 phenotype while maintaining CD161 expression (14). To test whether GM-CSF expression was associated with ex-Th17 cells, we determined levels of GM-CSF in CD161+ and CD161− Th1 cell subsets. This confirmed that only CD161+ Th1 cells were significantly enriched for GM-CSF expression compared to the total CD4+ population (Figure 2E). To investigate the transcriptional profile of CD161+ Th1 cells, SFMCs were sorted by cytokine capture into Th17 cells, CD161+ Th1 cells, and CD161− Th1 cells. CD161+ Th1 cells had significantly higher GM-CSF and RORC2 expression than CD161− Th1 cells, but equivalent levels of the Th1 transcription factor T-bet (Figure 2F).

**Figure 3:**
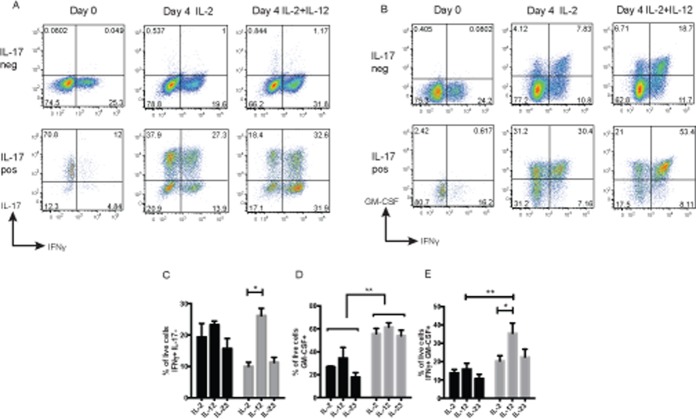
Transition of Th17 cells toward a GM-CSF–expressing phenotype in response to IL-12. A and B, IL-17+ and IL-17− CD4+ T cells from healthy control PBMCs were sorted by flow cytometry following cytokine capture and analyzed for expression of IFNγ and IL-17 (A) or IFNγ and GM-CSF (B) at baseline and after culture for 4 days in the presence of IL-2 (alone or with IL-12) prior to restimulation with phorbol myristate acetate and ionomycin in the presence of brefeldin A and analysis for cytokine expression. Results of a representative experiment (of 5 performed) are shown. C, IL-17+ CD4+ T cells (gray bars) and IL-17− CD4+ T cells (black bars) from healthy control PBMCs were cultured as described in A (as well as with IL-2 plus IL-23), and the proportion of live cells expressing IFNγ but not IL-17 was determined. D and E, IL-17+ CD4+ T cells (gray bars) and IL-17− CD4+ T cells (black bars) from healthy control PBMCs were cultured as described in B (as well as with IL-2 plus IL-23), and the proportion of live cells expressing GM-CSF (D) or IFNγ and GM-CSF (E) was determined. Values in C–E are the mean ± SEM. ∗ = *P* < 0.05; ∗∗ = *P* < 0.01, by two-way ANOVA with Bonferroni correction for multiple comparisons. See Figure 1 for definitions.

To test our hypothesis that Th17 cells undergo plasticity toward an IFNγ+GM-CSF+ phenotype, we used a cytokine capture assay to purify IL-17+ T helper cells and, as a control, IL-17− T helper cells, from healthy control PBMCs. Stimulation and culture of IL-17+ T cells in the presence of IL-2 down-regulated IL-17 and promoted plasticity toward an intermediate Th1/Th17 (IL-17+IFNγ+) and Th1 (IL-17−IFNγ+) phenotype (Figure 3A). We have previously demonstrated that IL-12 is significantly elevated in JIA SF compared to serum (14). To better understand the contribution of this cytokine to Th17 plasticity, we cultured cells in the presence of recombinant IL-12. This led to further differentiation toward Th1/Th17 and Th1 cells when compared to the results obtained after culture with IL-2 alone. IL-23, although important for murine Th17 plasticity, did not promote a Th1 phenotype (Figure 3C).

To track GM-CSF expression during Th17 plasticity, we stained cells for GM-CSF at baseline and after stimulation and culture in the presence of IL-12. At baseline, both the IL-17+ and the IL-17− populations exhibited minimal GM-CSF expression, whereas after activation, GM-CSF was up-regulated in IL-17+ cells significantly more than in IL-17− cells (Figures 3B and D). Most importantly, the IFNγ+GM-CSF+ T cell population identified in the joint was rapidly induced following culture of IL-17+ cells in the presence of IL-12 (Figure 3E). Our data demonstrate that plasticity toward a Th1 phenotype results not only in expression of IFNγ, but in concomitant up-regulation of GM-CSF expression.

## DISCUSSION

GM-CSF has long been recognized as an inflammatory mediator in the joints of patients with inflammatory arthritis. Although this has been extensively studied during the last 2 decades (9), recent data from murine models of autoimmunity have led to a reappraisal of the role of T cell–derived GM-CSF and its importance in arthritis. These data, together with the emergence of biologic agents targeting this cytokine, make the study of T cell GM-CSF expression and its regulation in human arthritis both timely and important.

In the present study we demonstrated an enrichment of GM-CSF–producing T helper cells in the joints of patients with JIA and, importantly, the frequency of these cells was directly correlated with levels of GM-CSF protein in the joint and serum markers of disease activity. To our knowledge, this is the first study to show that GM-CSF–expressing T cells in human autoimmune disease have a phenotype associated with ex-Th17 cells, coexpressing CD161 and IFNγ. These ex-Th17 cells, although lacking IL-17 expression, continue to express high levels of the Th17 transcription factor RORC2 (5,14). It is possible that RORC2 continues to play a role in the maintenance of GM-CSF within the ex-Th17 population. Expression of T-bet was not elevated in synovial T cells that were enriched for GM-CSF (Figure 2F), which is consistent with results of mouse studies showing normal GM-CSF expression in T-bet–knockout animals (6,7).

It has previously been shown that Th17 clones secrete GM-CSF (12), but, to our knowledge, this is the first study to demonstrate the role of IL-12 in driving Th17 plasticity toward a GM-CSF+ phenotype, and its possible role in arthritis. Interestingly, fate mapping studies have shown that IL-23, a member of the IL-12 cytokine family, has this effect in mice (16), while in humans IL-23 appears to stabilize the Th17 phenotype (17). The consequences of Th17 plasticity in arthritis were initially unclear, as the switch from IL-17 to IFNγ seemed unlikely to drive significant pathology given that administration of recombinant IFNγ to patients with rheumatoid arthritis has no adverse consequences (18). We propose that Th17 plasticity contributes to the persistence of joint inflammation by augmenting local levels of GM-CSF. The development of therapeutic antibodies targeting the GM-CSF receptor α-chain offers a valuable opportunity to test the importance of GM-CSF in JIA pathology, and early reports from rheumatoid arthritis studies are encouraging (19). Although our study has focused on Th17-related GM-CSF, Th17 cells are relatively rare in the joint and GM-CSF is also expressed by non–T cells, which suggests that Th17-independent GM-CSF could be important as well. Irrespective of the cellular source, receptor blockade would abrogate the downstream actions of GM-CSF and may be a viable therapeutic option in treatment-resistant JIA.

In conclusion, we have identified Th17 plasticity as a key driver of GM-CSF production within the arthritic joint. These results and the link between synovial GM-CSF enrichment and systemic inflammation strengthen the case for initiating trials of biologic agents that target the Th17 pathway, and in particular GM-CSF, in juvenile idiopathic arthritis.

## AUTHOR CONTRIBUTIONS

All authors were involved in drafting the article or revising it critically for important intellectual content, and all authors approved the final version to be published. Dr. Nistala had full access to all of the data in the study and takes responsibility for the integrity of the data and the accuracy of the data analysis.

**Study conception and design.** Pesenacker, Bending, Wedderburn, Nistala.

**Acquisition of data.** Piper, Pesenacker, Bending, Thirugnanabalan, Varsani, Nistala.

**Analysis and interpretation of data.** Piper, Pesenacker, Bending, Varsani, Wedderburn, Nistala.
